# Assessment of CA-125 First-Trimester Values as a Potential Screening Marker for Pre-Eclampsia

**DOI:** 10.3390/medicina59050891

**Published:** 2023-05-06

**Authors:** Oana Balint, Cristina Secosan, Laurentiu Pirtea

**Affiliations:** 1 Department of Obstetrics and Gynecology, University of Medicine and Pharmacy “Victor Babes”, 300041 Timișoara, Romania; secosan.cristina@umft.ro (C.S.);; 2 Emergency Clinical City Hospital Timișoara, Obstetrics-Gynecology Clinic, 300231 Timișoara, Romania

**Keywords:** CA-125, pre-eclampsia screening, pre-eclampsia markers

## Abstract

*Background*: Pre-eclampsia is a major public health issue. Current screening methods are based on maternal characteristics and medical history, but complex predictive models combining different clinical and biochemical markers have been proposed. However, although their accuracy is high, the implementation of these models in clinical practice is not always feasible, especially in low- and middle-resource settings. CA-125 is a tumoral marker, accessible and cheap, with proven potential as a severity marker in the third trimester of pregnancy in pre-eclamptic women. Assessment of its use as a first-trimester marker is necessary. *Methods*: This observational study involved fifty pregnant women between 11 and 14 weeks of pregnancy. Clinical and biochemical markers (PAPP-A), known for their value in pre-eclampsia screening, were recorded for every patient as well as first-trimester value of CA-125 and third-trimester data regarding blood pressure and pregnancy outcome. *Results*: No statistical correlation between CA-125 and first-trimester markers was observed except with PAPP-A, with which it exhibited a positive correlation. Additionally, no correlation was made between it and third-trimester blood pressure or pregnancy outcomes. *Conclusions*: CA-125 first-trimester values do not represent a valuable marker for pre-eclampsia screening. Further research on identifying an accessible and cheap marker to improve pre-eclampsia screening in low- and middle-income settings is needed.

## 1. Introduction

Currently, the most accepted pathophysiological theory regarding the development of pre-eclampsia suggests the presence of a two-stage process [1]. The first stage takes place in the first trimester of pregnancy and is a subclinical process. In the last decade, many researchers have focused on this stage and offered conditions for identifying effective strategies for the screening and/or prevention of pre-eclampsia.

The current screening approach for pre-eclampsia is based on the use of a combination of maternal characteristics and medical history to identify groups of high-risk women [2]. The National Institute for Health and Clinical Excellence (NICE) has defined several factors to stratify the risk of developing pre-eclampsia. According to their guidelines, a pregnant woman is considered to be at high risk if one of the following major factors are present: a personal history of hypertensive disease in any previous pregnancy, renal disease, diabetes mellitus, chronic essential hypertension or autoimmune disease; or if two of the following moderate factors are present: an age over 40 years, body mass index over 35 kg/m^2^, nulliparity family history of pre-eclampsia, or interpregnancy interval greater than 10 years [3]. However, although this approach is easy to apply in clinical practice, the evidence shows low detection rates of only 39% for pre-term pre-eclampsia and 34% for term pre-eclampsia [4].

Several different biochemical markers have been proposed as additional factors to the current approach to improve its detection rates. The most used markers are PAPP-A, PlGF and sFLT-1. CA-125 is a transmembrane glycoprotein and a member of the mucins group. Mucins are glycosylated in different manners and have functions dependent on the tissue in which they are found [5]. At the level of epithelial tissues, mucins provide protection against infection and tissue damage [6]. Several pathological processes, with secondary over-expression and glycosylation aberration, are known to cause changes in the structure and/or quantity of secreted mucins. These changes are reported in several cancers, thus explaining its use as a tumoral marker [5,7]. The dynamics of CA-125 levels in pregnancy are known, and increased values are reported in both the first trimester and immediately after birth, suggesting that the intense activity that takes place at the decidual level associated with those periods is a potential production origin [8,9,10]. Because pre-eclampsia is a placental pathology involving complex processes, CA-125 has been suggested as a possible marker in predicting pre-eclampsia severity. Several studies have analysed the use of CA-125 values in pre-eclampsia assessment [11,12,13,14,15]. A significant correlation between CA-125 values and the severity of pre-eclampsia was observed in all studies. Moreover, correlations with different clinical and biological parameters have also been reported, with positive correlations with mean arterial pressure, 24 h proteinuria, and LDH values and negative correlations with platelet number, gestational age at birth, and birth weight. Moreover, CA-125 values have shown significant correlations with IUGR and the rate of NICU admittance for new-borns [15]. In the only meta-analysis published on this subject, in 2019, diagnostic accuracy using CA-125 was reported at an AUC of 0.951, with a sensitivity of 91.9% and a specificity of 86.9%, for severe preeclampsia [16].

A valuable screening marker must identify asymptomatic individuals at sufficient risk for disease to justify therapeutic intervention to prevent or minimise the impact of the screened disease. Although the current screening approach can be applied in any clinical setting, its value is limited. The proposed clinical and biochemical markers and their combinations have demonstrated an increased detection rate, but none of the improved models have undergone clinical validation [17]. Moreover, screening using biomarkers, such as the sFLT-1/PlGF ratio or even measurement of the uterine artery Doppler pulsatility index (UtA-PI), can be challenging in low-resource settings.

Based on the existing research, CA-125 has the potential to be used as a first-trimester marker in pre-eclampsia screening. As a simple and affordable investigation, screening for pre-eclampsia in low-resource settings could be significantly improved if a predictive value could be demonstrated. Currently, there is no article published assessing its predictive value in the first trimester. This study was designed to analyse the relation between first-trimester CA-125 values and other known clinical and biochemical markers for pre-eclampsia as well as third-trimester blood pressure.

## 2. Materials and Methods

This observational study is part of a larger research project on CA-125 in the context of pre-eclampsia. It was led at a tertiary maternity facility of the Clinical Emergency City Hospital of Timisoara in the Obstetrics–Gynaecology Clinic between January 2022 and January 2023. The study population included pregnant women who required antenatal care in our hospital. The criteria for inclusion were pregnant patients between 11 + 0 and 14 + 6 weeks of gestation who were willing to attend complete follow-ups and provide the required data. The criteria for exclusion were refusal of inclusion in the study, refusal to attend complete follow-ups, chronic hypertension, chronic renal or hepatic disease, diabetes mellitus, a diagnosed gynaecologic pathology (ovarian or uterine disease), multiple pregnancies, diagnosed foetal anomalies, and pregnancies achieved by ART.

In the planned period for the study, fifty pregnant women were selected for inclusion, with five patients being lost at follow-up or from second-trimester spontaneous abortions. Using a standard interview, social and demographic characteristics, obstetric history, family history, and clinical data were obtained from every enrolled patient. Each patient’s PAPP-A, first-trimester mean arterial pressure, and uterine artery pulsatility index (UtA-PI) were converted in MoM. Blood samples for serum CA-125 were collected for every patient. The samples were processed using the ECLIA method (electrochemiluminescent immunoassay). The entire study population was given follow-up care until delivery. The hospital database was accessed for records of delivery, maternal, and neonatal outcomes.

For defining pregnancy-induced hypertension, two measurements of blood pressure obtained at least 4 h apart with values over 140 mmHg for systolic blood pressure, over 90 mmHg for diastolic blood pressure, or both and with appropriately sized cuffs were used. For further grouping of the hypertension group, criteria proposed by the American College of Obstetricians and Gynaecologists were used [18]. Pre-eclampsia was defined as a blood pressure measurement higher than 140 mmHg for systolic blood pressure, above 90 mmHg for diastolic blood pressure, or both, and the presence of a 24 h proteinuria of 300 mg/day. Severe pre-eclampsia was established when blood pressure measurements showed a systolic blood pressure over 160 mmHg, a diastolic blood pressure of 110 mmHg, or both from 2 measurements taken 4 h apart, and the presence of 24 h proteinuria over 500 mg/day. It was additionally established by the presence of headaches or visual disturbances, increased transaminase levels of at least twice their basal levels, upper abdominal pain, creatinine over 1.1 mg/dL, thrombocytopenia under 100.000/L, or pulmonary oedema.

Ethical approval for the current study was obtained from the Ethics Committee of the University of Medicine and Pharmacy, “Victor Babes,” Timisoara with No. 80/07.12.2020. All enrolled patients provided informed consent.

Jamovi software (The Jamovi Project [2021]. Jamovi [Version 1.6] [Computer Software]. Retrieved from https://www.jamovi.org) was used for statistical analysis. (Accessed on 23 August 2022) Descriptive statistics were applied for all appropriate data, and the normality of distribution was tested for all quantitative data. A *t*-test was performed to compare the two groups, and a one-way ANOVA test was applied for the parametric variables of multiple groups associated with the Tukey post-hoc test. Pearson’s correlation coefficient (Pearson’s r) was used to establish the correlation between different variables. A *p*-value of <0.05 was considered significant.

## 3. Results

Fifty patients were ultimately included for this study. The ages of the enrolled patients varied between 18 and 42 years old, with a mean of 29.3 years. Parity ranged between 0 and 3 births. The mean gestational age of patients at the moment of inclusion was 12.3 weeks.

When analysing the study population’s medical history, two of the included patients were found to have a history of pregnancy-related hypertension (any type). A total of 18% (n = 9) of the pregnant patients were smokers. Additionally, 20% (n = 10) of the pregnant patients took low-dose aspirin for pre-eclampsia prophylaxis. Indications were a history of pregnancy-related hypertension or age-related (over 35 years old) and nulliparity.

The mean PAPP-A value (MoM) was 1.41. A total of 12% (n = 6) of patients had a low level of PAPP-A (<0.4 MoM). The mean MAP was 86.3 mmHg (±10.2) or 1.01 MoM (±0.11). The mean UtAPI was 1.18 (±0.44) MoM. The mean CA-125 value was 20.2 UI/L (±11.8). When analysing correlations of the CA-125 first-trimester values with other parameters and markers used for pre-eclampsia first-trimester screening, a positive correlation was observed with PAPP-A values (MoM) (Table 1). Additionally, CA-125 values were not influenced by the age or parity of the patient.

In the third trimester, 12% of the pregnant patients (n = 6) had high blood pressure, with five patients diagnosed with gestational hypertension and one with mild pre-eclampsia. The mean MAP among patients with normal blood pressure was 96 mmHg (±8.1), while the mean MAP among patients with high blood pressure was 113 mmHg (±3.97) with a *p*-value < 0.001. The mean gestational age at birth was 38.3 weeks (±2.01), and the mean birth weight was 3223 g (±517). There were no statistical differences between patients with normal blood pressure and patients with high blood pressure regarding these parameters. When comparing first-trimester CA-125 values with third-trimester clinical parameters, no correlation was observed (Table 2).

The mean first-trimester CA-125 value of the patients with normal blood pressure in the third trimester was 16.6 UI/L (±8.78), while the mean first-trimester CA-125 value of the patients with third-trimester high blood pressure was 20.7 UI/L (±12.2). However, no statistical difference was observed with a *p*-value = 0.34.

## 4. Discussion

Our study investigated the probability of a correlation between CA-125 first-trimester levels and other first-trimester clinical or biochemical markers used in pre-eclampsia screening as well as with clinical third-trimester parameters, allowing us to identify a possible predictive marker that would improve the screening of pre-eclampsia.

Currently a few recognised markers are used for pre-eclampsia screening. PAPP-A (pregnancy-associated plasma protein A) is a regulatory protein secreted primarily by the syncytiotrophoblast that is essential for the bioavailability of the insulin-like growth factor used for normal foetal development. Initially used as an aneuploidy marker in first-trimester screening, it was later associated with unfavourable pregnancy outcomes such as intrauterine growth restriction, intrauterine foetal demise, premature birth, and pre-eclampsia [19,20]. Used as a single marker, its value is limited, with results below the 5th percentile (0.4 MoM) and giving an odds ratio between 1.5 and 4.6 for the occurrence of pre-eclampsia [21]. PlGF is a vascular endothelial growth factor predominantly expressed at the placental level, but it can also be identified in other human tissues. PlGF selectively connects to VEGFR-1 (vascular endothelial growth factor receptor 1), FLT-1 (fms-related tyrosine kinase-1), and its soluble variant sFLT-1. With this selectivity, PlGF is a proangiogenic marker, augmenting VEGF activity [22]. Research has shown that, in trophoblastic tissue, PlGF concentrations are diminished in the presence of hypoxia [23]. In pregnancies affected by pre-eclampsia, levels of PlGF have been shown to be low both at the time of diagnosis and in the advanced stages of the disease. In early pregnancy, the low concentration is caused by low expression as a result of abnormal placentation and the resulting hypoxia [24]. Used as a single marker, first-trimester PlGF dosing was associated with detection rates of only 55% for early and 33% for late pre-eclampsia, with a false-positive rate of 10% [25,26]. sFLT-1 is a protein tyrosine kinase with anti-angiogenic properties when attached to VEGF and PlGF [27]. The imbalance between pro-angiogenic and anti-angiogenic factors underlies the mechanism of pre-eclampsia. The association between the two markers, expressed as the sFLT-1/PlGF ratio, provided a natural starting point for identifying a superior predictive method. A ratio >85 for early pre-eclampsia and >110 for late pre-eclampsia was associated with a >95% sensitivity [28]. Another study identified a ratio cut-off of <38 with a negative predictive value of 99.3% to rule out pre-eclampsia in the following week [29].

Several studies have associated a high CA-125 value in the third trimester with severe pregnancy outcomes in pre-eclamptic patients [16]. The standardisation of its use could improve the identification of high-risk patients predisposed to disease progression and aid in decisions for hospitalisation, the moment of birth, or both. Moreover, CA-125 is affordable and widely available for investigation, and is, thus, a more accessible marker in low-resource settings where the management of pre-eclampsia is frequently suboptimal.

Pre-eclampsia represents one of the most researched topics in modern obstetrics. However, no cure has been found. Thus, its management is focused mainly on screening, prevention, early diagnosis, and reducing maternal and foetal morbidity and mortality to a minimum [30]. Current first-trimester screening recommendations are based on maternal characteristics and medical history [3]. High-risk women are then administered low-dose aspirin in order to prevent the onset of pre-eclampsia [31]. This screening method is associated with low detection rates. Several clinical or biochemical markers have been researched to improve detection rates. Although none showed significant predictive value as single markers, their combination developed predictive models with high accuracy [32]. The model proposed by The Fetal Medicine Foundation (FMF) combines clinical factors, maternal characteristics, MAP, UtA-PI, and PIGF and is the most valuable to date, being validated in a few large, multicentre trials [33,34].

It is well known that pre-eclampsia is a public health issue with a huge cost impact. Although the current prophylaxis method, aspirin, is quite cheap, effective screening models to establish the high-risk population that would benefit from it are complex and include some expensive investigations. The highest pre-eclampsia-related morbidity and mortality rates are reported in low- and middle-resource countries where most of the proposed screening methods are hard to implement successfully. Pre-eclampsia screening with basic tools, such as medical history, blood pressure measurements, or proteinuria dipstick detection, are the most appropriate and cost-effective methods. However, due to low detection rates, identifying additional cheap markers could significantly improve pre-eclampsia screening. Until identifying such markers, an alternative strategy that could be feasible even for low-resource areas is universal aspirin treatment. Arguments for this management method are both financial and medical. Aspirin has a good safety profile, with no increase in haemorrhagic complications [35]. It has been proven beneficial for high-risk women with a reduction of up to 80% (with good compliance) of pre-eclampsia development and has also been associated with improving functional placentation parameters even in low-risk pregnant patients when started in early pregnancy [36,37]. As for financial reasons, universal aspirin treatment was associated with the lowest costs compared to screening using biochemical and ultrasound markers, screening using patients’ characteristics and history, or not giving aspirin at all [38].

Based on the encouraging results reported in the third-trimester assessment with CA-125 as a severity marker for pre-eclampsia, this study analysed its potential use in the first trimester. The dynamics of CA-125 levels in pregnancy, including in the first trimester and with influencing factors, have been studied in several papers. Reported means of first-trimester values in healthy pregnant patients vary largely between 19.0 U/mL and 74.3 U/mL [39,40,41]. In our study, the mean value for CA-125 was 20.2 UI/mL (±11.8). All fifty included patients were healthy at the moment of enrolment. This difference could be attributed to race, a known factor affecting CA-125 values, as other patients’ characteristics were similar between study populations. As reported by others and in this study, patients’ ages or parity did not correlate with CA-125 levels. Additionally, levels higher than the used cut-off level of <35 U/mL (cut-off as a tumoral marker) in healthy, pregnant study population groups have been reported in only a few cases (<5) in each study. In our study, three patients had values over 35 U/mL.

Unfortunately, the results of this research show no correlation between CA-125 levels and some of the most used screening markers, except for PAPP-A, with which a positive correlation was observed. Moreover, first-trimester CA-125 values showed no correlation with blood pressure values measured in the third trimester for the normotensive pregnant patients or the pregnant patients with high-blood pressure. Additionally, no correlation was observed with pregnancy outcomes, such as gestational age at birth or birth weight.

## 5. Limitations

Significant limitations of this study are caused by the sample size of the study group, which is low as a result of hospital-based enrolment. Additionally, the absence of other studies focusing on the first trimester, the need for standardisation in CA-125 measurement, and the different biological and genetic characteristics of the populations studied in the third trimester impose caution in interpreting the results.

## 6. Conclusions

Pre-eclampsia remains a major public health issue due to its high maternal and foetal morbidity and mortality rates despite huge advances in knowledge over the last few decades. Current screening methods are either easily accessible for clinical practice but have low detection rates or have high accuracy but are extremely complex and expensive. CA-125 is a cheap and accessible marker, and its value in third-trimester screening has been proven. However, its first-trimester values do not correlate with other recognised pre-eclampsia screening markers or with third-trimester blood pressure values or pregnancy outcomes. Further research focusing on new methods that are accessible and cheap is needed, as improving the screening of pre-eclampsia, especially in low- and middle-income countries, is a topic that must be treated with high priority.

## Figures and Tables

**Table 1:** Correlations of first-trimester CA-125 values with different markers used for pre-eclampsia screening.

Parameter	Pearson’s Correlation Coefficient	*p*-Value
Age	−0.220	0.125
Parity	−0.080	0.582
Gestational age	0.054	0.710
PAPP-A (MoM)	0.390	0.005 *
MAP (MoM)	0.042	0.77
UtA-PI (MoM)	0.124	0.39

* Statistically significant result.

**Table 2:** Correlations between first-trimester CA-125 values and different third-trimester clinical parameters.

Parameter	Pearson’s Correlation Coefficient	*p*-Value
T3 MAP	0.176	0.22
Gestational age at birth	−0.053	0.71
Birth weight	−0.134	0.35

## Data Availability

Data are available from the first author upon reasonable request.
